# Refocusing on the foundations: strategy for child and adolescent health in Europe and Central Asia 2026–2030 – A healthy start for a healthy life

**DOI:** 10.7189/jogh.15.03046

**Published:** 2025-10-30

**Authors:** Sophie Jullien, Ivelina Borisova, Joao Breda, Susanne Carai, Gabriele Fontana, Aleksandra Jovic, Martin M Weber, Octavian Bivol, Natasha Azzopardi Muscat

**Affiliations:** 1 WHO Office for Quality of Care and Patient Safety, WHO Regional Office for Europe, Athens, Greece; 2 UNICEF Europe and Central Asia Regional Office, Geneva, Switzerland; 3 WHO Regional Office for Europe, Copenhagen, Denmark

## Abstract

The World Health Organization (WHO) Regional Office for Europe and the United Nations Children’s Fund (UNIICEF) Europe and Central Asia have jointly led the development process of a new strategy for children and adolescents in Europe – a healthy start for a healthy life – to guide evidence-based, future-oriented action across the Region. It was developed through a multi-step process, including a regional assessment, commissioned evidence reviews, and a multi-stakeholder survey. Consultations with Member States, technical experts, and adolescents ensured a participatory approach. The strategy identifies five areas for action: investing in child and adolescent health, ensuring access to high-quality care, protecting against commercial and digital harms, fostering multisectoral collaboration, and strengthening accountability through improved data and monitoring. The strategy provides clear roles for countries and partners and is designed to support equitable, evidence-based implementation across the WHO European Region. It introduces an innovative framework that addresses both persistent gaps and emerging threats. Its dual-level structure, with defined roles for both Member States and WHO/UNICEF, reflects a more collaborative and accountable model of implementation. The emphasis on adolescent participation, regulatory action, and multisectoral investment signals a shift toward more inclusive and systemic approaches to child and adolescent health and well-being. To succeed, the strategy will require strong political will, sustained investment, and active engagement from health professionals, communities, and children and adolescents themselves.

Europe and Central Asia are failing their children [1]. Over five million children are at risk of difficulties in learning, development, and daily functioning [2]. Mental health issues are on the rise, disproportionately affecting children from socioeconomically disadvantaged backgrounds [3]. The region also continues to report the lowest breastfeeding rates globally and a growing burden of childhood obesity, now affecting one in three primary school-aged children and one in four adolescents [4,5]. The digital environment has introduced new risks, with over 10% of teenagers exhibiting problematic social media use [6]. The health and well-being of children and adolescents in the World Health Organization (WHO) European Region are at a critical juncture.

The previous WHO strategy for child and adolescent health, which guided Regional efforts from 2015 to 2020, provided a foundational framework for improving health outcomes across the life course [7]. It has led to the development of national child and adolescent health strategies in more countries, with increased youth involvement in shaping policies [8]. However, progress was uneven and significant gaps remain, particularly in addressing mental health, health equity, and the broader determinants of health, and emerging challenges such as digital health disparities and the growing burden of non-communicable diseases [8–11].

The convergence of multiple and ongoing crises, including armed conflicts and the escalating climate emergency, has significantly diverted political attention and resources away from child and adolescent health [1,12]. These overlapping emergencies have reshaped policy priorities, threatening the progress made toward a holistic, lifecycle-based approach to health [13]. The COVID-19 pandemic exacerbated this setback. Although children were less directly affected by the virus itself, they bore a disproportionate burden of the infection control measures, such as school closures and social isolation. The long-term consequences of this lost focus, particularly the impact of pandemic-related policies, are expected to reverberate for decades, potentially undermining developmental, educational, and psychosocial outcomes for an entire generation.

Against this backdrop and in response to the urgent challenges, the WHO Regional Office for Europe and the United Nations Children’s Fund (UNICEF) Europe and Central Asia (ECARO) have jointly led the process of development of a new strategy for child and adolescent health and well-being (CAHW). This strategy represents a paradigm shift – moving beyond vertical, disease-specific approaches to embrace a holistic, rights-based, and cross-sectoral vision of health and well-being. It is grounded in the epidemiological, social, and policy realities of the Region, and reflects a shared commitment to ‘future-proof’ the health of children and adolescents across Europe and Central Asia [1]. In their joint letter, the Regional Directors of WHO Regional Office for Europe and UNICEF ECARO emphasised the need for bold, evidence-based action to address both longstanding and novel threats to child and adolescent health [1].

Here, we describe the steps taken in the development of the new CAHW strategy, ‘A healthy start for a healthy life’, including the commissioning of a series of thematic reviews to better understand the challenges facing children and adolescents in the Region. We present the main findings of these reviews and outline the core content and priorities of the new strategy.

## CO-DESIGNING THE CAHW STRATEGY: EVIDENCE, ENGAGEMNT, AND CONSULTATION

### Assessment of the current situation

To inform the core content and proposed actions of the CAHW strategy, we conducted a comprehensive assessment of the current health status and challenges faced by children and adolescents in the region. A Member State survey was carried out, complemented with relevant data from other available sources and findings summarised in the WHO Progress Report on Child and Adolescent Health in Europe, which provides updates on achievements and the status of health and well-being of children and adolescents to 2021 – corresponding to the end of the 2015–20 strategy – with insights into key trends, gaps, and priority areas [8].

### Commissioned evidence reviews

The WHO commissioned a series of thematic reviews to support the development of evidence-based actions within the strategy [14]. These reviews synthesise current global and regional evidence across key areas relevant to child and adolescent health priorities, including child and adolescent mortality, impact of the pandemic restrictions on early childhood development, impact of school closures on educational outcomes and adolescent well-being, changing determinants of mental health, obesity and digital determinants, and the health of forcibly displaced children. The main findings of these evidence reviews are summarised elsewhere [15].

A series of thematic fact sheets were also developed to further support the translation of this evidence into action, highlighting key messages and policy implications for each priority area [16]. These resources aim to facilitate stakeholder engagement and inform national and regional implementation efforts.

### Multi-stakeholder survey

The WHO Regional Office for Europe and UNICEF ECARO conducted an additional regional survey targeting a diverse group of stakeholders, including Member State official representatives (child and adolescent focal points appointed by the relevant Ministry of Health), child health professionals, adolescents, and parents of young children. The survey aimed to identify priority areas for CAHW, as well as the types of technical support required for countries to address these priorities effectively. Participants were invited to assess a series of problem statements and support needs related to CAHW using a five-point scale to indicate their level of concern. The findings of this survey are provided elsewhere [17].

### Member States consultations

In the co-creation exercise of developing the strategy, three rounds of consultations were held with the Member State official representatives between November 2024 and April 2025, conducted in both virtual and in-person formats. These consultations served multiple purposes: validating the identified priorities, gathering input on strategic directions, and collaboratively developing and refining proposed actions for countries as well as for WHO/Europe and UNICEF. Draft versions of the strategy were circulated between consultation rounds, and written feedback received from Member States was systematically reviewed, incorporated, and discussed during the meetings, ensuring a transparent and inclusive development process.

### Advisory group

To support the co-creation of the strategy and ensure it reflected country-level priorities, a writing advisory group was established following the first Member States consultation. Official representatives of five countries – Armenia, Croatia, Portugal, the Russian Federation, and Spain – volunteered to participate, forming a diverse and representative group from across the WHO European Region. The group aimed to co-create and review the different iterations of the draft strategy for CAHW, providing structured input on several key areas: overall strategy coherence, alignment with national priorities, identification of critical issues to be addressed, potential barriers to implementation, and suggestions for feasible implementation approaches. Routine meetings were held with representatives from the WHO and the UNICEF, and were chaired by Public Health Scotland, a WHO Collaborating Centre. The group’s feedback was instrumental in shaping a strategy that is both regionally relevant and practically actionable.

### Adolescents’ participation

Targeted engagement activities were conducted with adolescents across the region in the different steps of the strategy development, including participatory workshops and focus group discussions. Adolescent representatives also took part in the in-person consultation held in 2025, where they shared their perspectives and reflections from the focus group discussions led by youth advisory councils. The multi-stakeholder survey described above was also conducted through adolescent networks to ensure their perspectives were meaningfully integrated into the strategy.

### Technical collaboration

The development process was supported by WHO Collaborating Centres, which provided technical expertise, contributed to evidence synthesis, and supported the design of strategic components.

### Synthesis and strategy development

Findings from all sources were synthesised through iterative internal meetings and expert consultations. This collaborative process informed the development of the strategy’s vision, strategic priorities, and implementation pathways. The resulting framework outlines targeted actions for countries as well as for the WHO and the UNICEF, recognising the shared responsibility in advancing child and adolescent health and well-being across the region. The dual-level approach ensures that both national and regional actors are equipped to contribute effectively to the strategy’s vision.

## A HEALTHY START FOR A HEALTHY LIFE – A STRATEGY FOR CHILD AND ADOLESCENT HEALTH AND WELL-BEING IN THE WHO EUROPEAN REGION 2026-2030

### Key challenges in child and adolescent health and well-being

**Figure 1:**
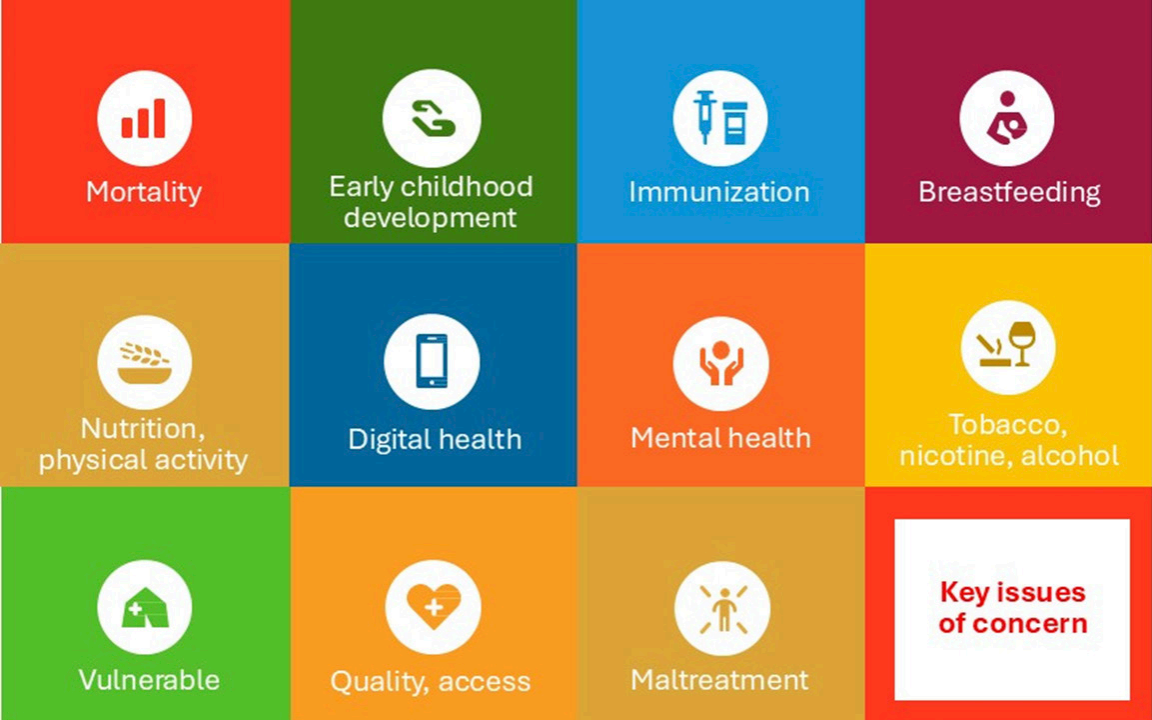
Key issues of concern for children and adolescents in the WHO European Region.

Children and adolescents in the WHO European Region face a growing array of health challenges (**Figure 1**), many of which are preventable or manageable with timely, high-quality care. Progress in reducing mortality has stalled, with some countries seeing reversals, particularly among neonates and adolescents. Access to child- and adolescent-friendly health services remains inconsistent, while primary healthcare is still underutilised. Early childhood development is at risk for millions, while immunisation rates are declining and breastfeeding remains low. Overweight, obesity, and poor oral health are widespread, especially among vulnerable groups. Digital technologies, while offering benefits, are contributing to mental health issues and problematic behaviours. Substance use, violence, and the needs of children in vulnerable situations – including displaced and socioeconomically disadvantaged populations – further compound these challenges, demanding urgent, coordinated action.

Efforts to prevent non-communicable diseases and promote healthy ageing that begin only in adulthood are fundamentally flawed. Just as no building can be stable without a strong foundation, no population can age healthily if the critical developmental stages of childhood and adolescence are neglected. This is when lifelong behaviours, resilience, and risks are shaped.

The current trajectory of the development of health and well-being of children and adolescents in Europe and Central Asia needs to be corrected to secure the future prosperity and resilience of societies and economies.

### Strategic framework and priority areas for action

**Figure 2:**
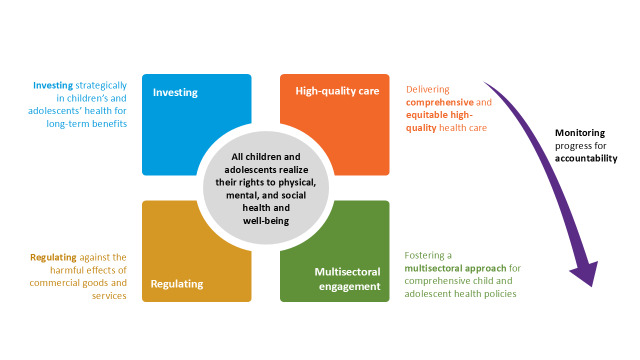
The five priority areas for action of the WHO/UNICEF child and adolescent health strategy for the European Region.

In response to the above key challenges, the WHO Regional Office for Europe and UNICEF ECARO led the development process of a joint strategy for 2026–30, grounded in the principles of equity, rights, and multisectoral collaboration. The strategy outlines a vision in which every child and adolescent in the Region can thrive in healthy, supportive environments. It proposes five priority areas for action, each with specific roles for both countries and international partners: strategic investment in child and adolescent health and well-being; delivery of comprehensive and equitable high-quality healthcare; regulation to protect against commercial harm; fostering multisectoral collaboration; and monitoring progress for accountability (**Figure 2**).

### Strategic investment in child and adolescent health and well-being

This area emphasises the need for sustained and equitable investment in child and adolescent health as a foundation for long-term societal and economic benefits. Following progress under the Millennium Development Goals (MDGs), investment declined during the era of Sustainable Development Goals (SDGs) due to the perception that challenges had been resolved. The results of this disinvestment and de-prioritisation are now clearly seen in the data and must be corrected. Countries are encouraged to increase public spending on health services for children and adolescents, ensure full cost coverage for essential services, and adopt equity-weighted budgeting models. Investments should also target workforce development, including adequate staffing, fair remuneration, and continuous professional training aligned with international standards. Beyond the health sector, multisectoral investments are needed in areas such as education, parenting support, and healthy environments. The WHO and the UNICEF will support countries through advocacy, technical assistance, and capacity-building, particularly in low-resource and conflict-affected settings.

### Delivery of comprehensive and equitable high-quality healthcare

This priority area seeks to ensure that all children and adolescents have access to quality, age-appropriate health services without financial hardship. Countries are encouraged to develop or update national evidence-based standards of care, strengthen primary healthcare, as well as referral and collaboration with specialist services, and improve long-term and rehabilitative services. Key interventions include promoting evidence-based healthcare in the best interest of the child or adolescents, including vaccination, breastfeeding, mental healthcare, and early childhood development, as well as addressing violence, substance use, and the transition to adult care. The WHO and the UNICEF will assist in adapting and implementing evidence-based guidelines and frameworks, and in building capacity to deliver integrated, person-centred care across the life course.

### Regulation to protect against commercial harm

Recognising the growing influence of commercial determinants of health, this area calls for stronger regulation to limit the marketing of unhealthy foods and beverages – particularly those high in fat, salt, and sugar – as well as breast milk substitutes, in line with international codes and guidance. It also calls for robust regulation of tobacco, nicotine, and alcohol products, including through taxation, age restrictions, and marketing bans, to reduce accessibility and appeal among young people. Countries are urged to implement comprehensive policies to restrict marketing – especially digital marketing – to children and adolescents, mandate healthy food environments in schools, and use fiscal measures to reduce the affordability of harmful products. Protection from digital harms, including algorithmic manipulation and data exploitation, is also emphasised. The WHO and the UNICEF will provide model legislation, regulatory toolkits, and platforms for sharing best practices across Member States.

### Fostering multisectoral collaboration

Improving child and adolescent health requires coordinated action across sectors. This area promotes the development of national strategies that integrate health with education, social protection, urban planning, and other domains. Countries are encouraged to establish cross-sectoral coordination mechanisms such as cross-ministry units, invest in joint training, and implement family-friendly policies. The strategy also supports the expansion of health-promoting schools, integration of health education into curricula, and outreach to out-of-school adolescents. The WHO and the UNICEF will assist in operationalising multisectoral strategies, supporting implementation of global standards, and facilitating knowledge exchange across the Region.

### Monitoring progress for accountability

Robust monitoring systems are essential to track progress and ensure accountability. Countries are called to strengthen health information systems, collect disaggregated data, and report on key indicators through international mechanisms such as the Health Behaviour in School-aged Children (HBSC) study, the Global Youth Tobacco Survey (GYTS), the Childhood Obesity Surveillance Initiative, and the Global Action for Measurement of Adolescent Health. The strategy encourages the development of public dashboards and the inclusion of underrepresented groups in data collection. The WHO and the UNICEF will support countries in building digital infrastructure, developing unified monitoring frameworks, and establishing a Regional Youth Advisory Board to ensure youth participation in strategic reviews. Progress will be reviewed at mid-term (2027) and end line (2030).

## WHAT IS NEW AND WAY FORWARD

The development of the new strategy for CAHW by the WHO Regional Office for Europe and UNICEF ECARO comes at a pivotal moment for the Region. Despite decades of progress, recent trends indicate that Europe and Central Asia are failing to meet the evolving needs of their youngest populations. The Region is facing a convergence of persistent and emerging challenges, including the stagnation – and in some cases reversal – of child and adolescent mortality rates, rising mental health concerns, and widening health inequities, underscoring the urgency for a renewed, comprehensive, and forward-looking approach.

While the Global Strategy for Women’s, Children’s, and Adolescents’ Health provides a comprehensive framework for improving health outcomes across the life course, its global scope does not fully capture the specific epidemiological, social, and policy contexts of the WHO European Region. There remains a critical need for a strategy tailored to the unique challenges and contexts of the WHO European Region [18]. Although 76% of countries in the Region had adopted a national strategy for children and adolescents within the previous five years, 16% were still in the process of doing so, and 9% had no such strategy in place [8]. A dedicated European CAHW strategy offers a timely and targeted response to these gaps: it not only aligns national efforts with global priorities, but also addresses region-specific issues such as demographic shifts, health inequities, and the compounded effects of recent crises on young populations.

The previous WHO European Strategy for Child and Adolescent Health (2015–2020) laid a strong foundation by promoting a life-course approach, strengthening early childhood development, and addressing preventable mortality and morbidity [7]. It emphasised intersectoral collaboration, rights-based principles, and the need to make children’s lives visible in policy and data. However, while the strategy catalysed improvements in national policy frameworks and service delivery in several countries, its impact was uneven. Persistent gaps in mental healthcare, immunisation coverage, and access to adolescent-friendly services remained unaddressed in many settings. Moreover, as threats are developing, new issues become prominent, such as digital health risks, climate-related vulnerabilities, and the growing burden of non-communicable diseases among children and adolescents.

The new CAHW strategy (2026–2030), titled ‘A healthy start for a healthy life’, builds on the legacy of its predecessor while introducing several critical innovations. First, it is explicitly designed to respond to the current European and Central Asian context, marked by geopolitical instability, economic uncertainty, and the long-term impacts of the COVID-19 pandemic. These factors have exacerbated existing inequities and placed additional strain on health systems, particularly in low-resource and conflict-affected countries. The strategy acknowledges these realities and proposes differentiated support for countries based on their specific needs and capacities.

Second, the strategy adopts a dual-level approach, outlining concrete actions for both Member States, the WHO, and the UNICEF. This reflects a shift from a purely country-driven model to one that recognises the shared responsibility of regional and global actors in supporting national implementation. For example, while countries are encouraged to invest in child health services and workforce development, the WHO and the UNICEF commit to providing technical assistance, model legislation, and platforms for knowledge exchange. This co-accountability model is particularly important in a region as diverse as the WHO European Region, where health system capacities and political priorities vary widely.

Third, the strategy places a stronger emphasis on the commercial and digital determinants of health. While the 2015–20 strategy addressed tobacco and alcohol use, the new framework expands regulatory focus to include the marketing of unhealthy foods, breast-milk substitutes, and digital harms such as algorithmic manipulation and data exploitation. This is a timely and necessary evolution, given the increasing influence of digital platforms on children’s behaviours, mental health, and exposure to harmful content [6]. The strategy’s call for comprehensive regulation – backed by WHO-provided legal toolkits and monitoring frameworks – marks a significant advancement in protecting children from commercial exploitation.

The strategy was jointly developed by the WHO Regional Office for Europe and the UNICEF ECARO, leveraging the complementary mandates and expertise of both agencies. The WHO brings leadership in public health, health systems strengthening, and normative guidance, while the UNICEF contributes deep experience in child rights, education, and social protection. This collaboration ensured a multisectoral and rights-based approach, grounded in both health and broader well-being dimensions. The joint leadership also reinforces the shared accountability of UN agencies in supporting Member States to implement the strategy effectively.

Another key innovation is the strategy’s commitment to meaningful youth engagement. Children and adolescents were consulted throughout the development process, and the strategy proposes the establishment of a Regional Youth Advisory Board to participate in monitoring and strategic reviews. This aligns with the principles of the United Nations Convention on the Rights of the Child, which enshrines the right of young people to participate in decisions affecting their lives [19]. It also reflects growing recognition that youth engagement is not only a moral imperative but also a practical necessity for designing effective, relevant policies.

A critical factor in the successful implementation of the CAHW strategy is the sustained engagement of child and adolescent health professionals. These practitioners – paediatricians, nurses, psychologists, school health staff, and community health workers – are not only the frontline providers of care but also key advocates for children’s rights and well-being. Their insights are essential for translating policy into practice, identifying emerging challenges, and tailoring interventions to local contexts. The strategy rightly emphasizes the need for continuous professional development, adequate remuneration, and workforce planning to ensure a motivated and skilled health workforce. Moreover, involving health professionals in the design, monitoring, and evaluation of national strategies fosters ownership and accountability. Their active participation can also help bridge gaps between health systems and communities, ensuring that services are accessible, acceptable, and responsive to the needs of children and adolescents.

The strategy’s five priority areas – strategic investment, high-quality healthcare, regulation, multisectoral collaboration, and monitoring – are comprehensive and interdependent. Together, they address both the immediate and structural determinants of child and adolescent health. For instance, the emphasis on early investment and equity-weighted budgeting models is grounded in evidence showing that early interventions yield high returns in terms of educational attainment, economic productivity, and reduced healthcare costs. Similarly, the focus on multisectoral collaboration acknowledges that health outcomes are shaped by policies in education, urban planning, social protection, and beyond.

Importantly, the strategy also addresses long-standing data gaps that have hindered progress in the past. By encouraging countries to strengthen health information systems, disaggregate data by equity metrics, and participate in international monitoring initiatives such as the HBSC study and the GYTS, the strategy lays the groundwork for more transparent, accountable, and evidence-informed policymaking.

As the global health community moves towards the post-SDG era, there is growing recognition of the need to refocus attention on children and adolescents – groups that have risked becoming invisible within broad, system-oriented agendas. While universal health coverage rightly remains a central pillar of global health, its implementation must be balanced with targeted investments and accountability mechanisms for this specific population group. During the MDG era, explicit goals related to child survival helped mobilise political will and resources. In contrast, the more diffuse nature of the SDGs has diluted focus and fragmented action for children and adolescents. A renewed commitment is needed – one that aligns with the principles of equity and systems strengthening, yet acknowledges that progress for children and adolescents requires both comprehensive and intentional approaches. Without these other global health targets, such as healthy ageing, a reduced burden of non-communicable diseases, and mental health improvements, will remain out of reach.

Despite its strengths, the strategy’s success will depend on several factors. First, political will and sustained investment are essential. In a resource-constrained environment, governments may face competing priorities, and child health may not always receive the attention it deserves. Advocacy efforts – supported by the WHO and the UNICEF – will be critical to ensure that CAHW remains high on the political agenda. Second, implementation capacity varies widely across the Region. Countries with weaker health systems may require intensive technical and financial support to operationalise the strategy’s recommendations.

## CONCLUSIONS

The new CAHW strategy represents a bold and timely response to the evolving needs of children and adolescents in the WHO European Region. It builds on past achievements, while addressing critical gaps and emerging threats. By combining evidence-based interventions with a strong equity lens and by fostering shared responsibility across sectors and stakeholders, the strategy offers a roadmap for transforming child and adolescent health in Europe and Central Asia. Its success will depend not only on the strength of its design, but also on the commitment of governments, partners, and communities to turn vision into action.
